# A New Model to Investigate the Action of Radiation and Cigarette Smoke on Head and Neck Cancer Cells

**DOI:** 10.3390/cancers17081346

**Published:** 2025-04-17

**Authors:** Kylie Lopes Floro, Rhys Gillman, Miriam Wankell, Brittany Dewdney, Madhavi Chilkuri, Ashley Shackelford, Leslie Kuma, Marcus Powers, Lionel Hebbard

**Affiliations:** 1 Radiation Oncology, Townsville University Hospital, Townsville, QLD 4814, Australia; 2 Centre for Molecular Therapeutics, Department of Molecular Genetics, Australian Institute of Tropical Medicine and Health, College of Medicine and Dentistry, James Cook University, Townsville, QLD 4811, Australia; 3 Radiation Oncology, Cancer Care Services, Royal Brisbane and Women’s Hospital, Herston, QLD 4006, Australia; 4 The Kids Research Institute of Australia, Nedlands, WA 6009, Australia; 5 Radiation Oncology, University Hospital Geelong, Geelong, VIC 3220, Australia; 6 Capital Pathology, Equinox 4, Ground Floor, 70 Kent St., Deakin, ACT 2600, Australia; 7 Cancer Care Noosa, 36-40, Hofmann Drive, Noosaville, QLD 4566, Australia; 8 Storr Liver Centre, Westmead Institute for Medical Research, Westmead Hospital and University of Sydney, Sydney, NSW 2145, Australia

**Keywords:** radiation, smoking, head and neck cancer

## Abstract

**Background/Objectives**: Smokers are at an increased risk of developing mucosal head and neck squamous cell cancers (HNSCCs) and have a worse prognosis when treated. The cellular and molecular mechanisms underlying the latter has not been established. We therefore developed an in vitro model to investigate the effects of radiation and smoking on mucosal HNSCCs. FaDu hypopharyngeal cancer cells were subjected to daily fractionated radiation and cultured with and without cigarette-smoke-exposed media. **Methods**: The cells were characterised using assays for tumour sphere formation, proliferation, migration, invasion, CD44 and ALDH expression, and next generation sequencing. We also evaluated CD44 and ALDH1 expression in patient tumour samples. **Results**: Radiation and smoking separately reduced FaDu tumour sphere/Cancer Stem Cell (CSC) number and proliferation, and increased cell migration and invasion. Combined, they further reduced CSC number proliferation and promoted migration. CD44 and ALDH co-expression was reduced in conditions with cigarette smoke. Through next generation sequencing, radiation and smoking produced a gene signature related to cell invasion, angiogenesis, and survival. Immunohistochemistry for CD44 and ALDH1 on patient tumour specimens did not demonstrate a relationship with smoking status, supported our in vitro findings. **Conclusions**: The data show the utility of a new experimental model to test the combination of radiation and smoking on mucosal HNSCCs behaviour.

## Simple Summary

Smokers are at an increased risk of developing mucosal head and neck cancers. Moreover, they have worse oncological outcomes following treatment; the reasons for this are unknown. To address this, we developed a new experimental model investigating the effects of radiation and smoking on head and neck cancer cells. We found through that radiation and smoking separately altered cell behaviour, and when combined had a greater effect. Gene sequencing reflected the changes in cell behaviour after treatments. Our results show that this new experimental model is relevant in evaluating the combination of radiation and smoking on head and neck cancer cell behaviour.

## 1. Introduction

In 2020, head and neck squamous cell cancers (HNSCCs) were the eighth most common tumour, with over 900,000 cases worldwide [1]. Mucosal HNSCCs arise in the mucosa of the oral cavity, pharynx, larynx, nasal cavity, and paranasal sinuses. The Human Papilloma Virus (HPV), smoking, and alcohol use are risk factors for mucosal SCCs and influence prognosis. The 5-year survival for HNSCC varies widely with tumour site and stage. For example, early-stage hypopharyngeal disease has a survival rate of less than 60%, and more advanced disease has a survival rate of less than 25% [2]. HNSCCs are aggressive invasive cancers and management includes curative surgery, radiation therapy, or a combination of both, with and without chemotherapy [3].

HPV-positive HNSCCs are predominantly oropharyngeal cancers that are more sensitive to chemoradiation therapy and have better locoregional control and progression-free and overall survival than HPV-negative cancers [4,5]. Nonetheless, the continuation of smoking after the diagnosis of head and neck cancer decreases locoregional control and disease-free and overall survival [6,7,8]. Consistent with this, smokers with HPV-positive cancers appear to have an intermediate prognosis similar to HPV-negative cancers from non-smokers [5,7]. Molecular changes likely underpin the prognostic differences seen in HNSCC exposed to HPV and cigarette smoke.

The cancer stem cell hypothesis postulates that a subpopulation of cells within a tumour can self-renew and proliferate extensively, thus driving tumourigenesis. These cells are thought to be the cause for progression, metastases, and post-treatment recurrence and have been identified as cancer stem cells (CSCs) in several tumours including HNSCC. Given poorer outcomes for smokers with HNSCC, cigarette smoke likely alters cell behaviour and function. Nicotine exposure of primary oral squamous cell carcinoma cells has been shown to induce markers of epithelial-to-mesenchymal transition (EMT) and stemness [9]. Thus, cigarette smoke, which contains nicotine, in addition to other constituents, may promote a similar effect in HNSCC cells. Likewise, radiation therapy can cause HPV-negative cancer cell lines to increase de-differentiating properties in vitro and form more CSCs versus HPV-positive cancers [10,11]. This may in part explain the poorer prognosis of HPV-negative cancers. While the individual effects of radiation and nicotine have been evaluated in HNSCC cells, no study has examined the combined action of radiation and smoking (RS) in vitro on HNSCCs and how this effects cell behaviour and gene expression.

HNSCC CSCs can be identified by the isolation of sphere-forming cells (tumour spheres) as a marker of self-renewal, and the expression of stem cell markers such as CD44 and ALDH1 [12,13]. CD44 is a transmembrane glycoprotein expressed by CSCs and is prognostic for HNSCC recurrence [14,15,16,17]. ALDH1 is a cytosolic enzyme that reduces reactive oxygen species and can identify CSCs in HNSCCs and predict poor prognosis and metastasis [11,18,19,20,21,22]. While CD44 and ALDH1 can identify CSCs, it was found that HNSCC cells having dual expression of both CD44 and ALDH1 HNSCC cells have greater tumourigenic and radiation-resistant attributes and may associate with tumour proclivity [23]. Studies have also shown an increase in the number of CD44/ALDH-expressing cells when treated with radiation [10,11]. Thus, we studied the effect of conventional fractionated radiation therapy and cigarette smoke in vitro and evaluated molecular changes. Additionally, given that the proportion of CSCs in untreated tumours could vary greatly between smokers and non-smokers, and correlate with poor prognosis, we assayed for CD44 and ALDH1 co-expression prior to radiation therapy.

## 2. Materials and Methods

### 2.1. Cell Culture

The HPV-negative cancer cell lines FaDu (Pharynx; ATCC^®^ HTB-43^TM^) and SCC15 (Tongue, ATCC^®^ CRL-1623) were obtained from Associate Professor Fiona Simpson of the Queensland Head and Neck Research Centre. Cells were cultured using standard procedures at 37 °C in a humidified incubator with 5% CO_2_ in DMEM-F12 (Sigma Aldrich, St Louis, MO, USA) supplemented with 10% foetal calf serum (Corning 35-076-CV, Corning, NY, USA) and 1% penicillin and streptomycin (Sigma Aldrich). Using negative pressure, DMEM-F12 was exposed to cigarette smoke and particulate matter; 25 John Player and Sons (JPS) Red label cigarettes were used per 500 mL of DMEM-F12. Each cigarette took approximately 4 min to burn, and the media were filtered (0.22 mm) and stored at minus 80 °C. Cells were treated with cigarette-exposed media in a 4-day cycle of a 1/50 dilution for two days, followed by a 1/25 dilution for two days. The cycle was repeated for the duration of the experiment until the cells were subjected to specific assays.

### 2.2. Cell Irradiation

Cells were grown to 80% confluency and irradiated in T75 flasks (Greiner, Kremsmünster, Austria) within a wax chamber. Using an Elekta linear accelerator, a 6 MV photon beam was delivered to the chamber at 1.8 Gy/fraction, and 5 fractions per week for 3 weeks. These values were chosen to emulate clinical treatment. Prior to treating cells, the radiation plan underwent quality assurance using film dosimetry with Gafchromic EBT3 film (Ashland™, Wilmington, DE, USA). There was <1% difference between the treatment planning system-predicted dose and the dose delivered; this was within the known expected uncertainty of film dosimetry. For next generation sequencing and flow cytometry assays, cells were cultured for 3 days following irradiation prior to use, and until confluent for migration assays.

### 2.3. MTT Cell Viability Assay

A total of 4000 cells per well were seeded in 96-well plates in replicates of 6 and treated for 24–48 h with media containing cigarette smoke extract, after which 3-(4,5-Dimethylthiazol-2-yl)-2,5-Diphenyltetrazolium Bromide (MTT) (Sigma Aldrich, M5655) was supplied to each well for 2 h (final concentration; 0.5 mg/mL), the media was removed, DMSO was supplied, and the absorbance was read (540 nm; FLUOstar Omega microplate reader, BMG LabTech, Offenburg, Germany).

### 2.4. Tumour Sphere Assay

A total of 2 × 10^4^ cells/well was supplied to 6-well low-attachment plates (Sigma Aldrich; 3 replicates) in media (2% B27 Supplement (ThermoFisher, Waltham, MA, USA), 20 ng/mL hEGF (Sigma Aldrich), and 10 ng/mL basic fibroblast growth factor (bFGF; Thermofisher)). Cigarette smoke extract was diluted in media 1/25 and spheres were counted after 5 days.

### 2.5. Wound Healing Assay

An amount of 2 × 10^5^ cells/well was seeded in 6-well plates in triplicate in media with or without cigarette smoke diluted in media 1/25 and cultured until confluent. A wound was made to the confluent layer of cells using a 200 mL pipette tip, images of the healing confluent layer were taken with an inverted microscope, and using ImageJ (Version 1.54) the distance migrated in µm/hour was determined.

### 2.6. Invasion Assay

The upper surface of 6.5 mm Transwell filters (8.0 µm pore, Corning 3422) was coated with 50 μL of Corning Matrigel and 5 × 10^5^ irradiated or non-irradiated cells were seeded into the upper chamber in serum-free media with/without cigarette smoke extract at a 1/25 dilution. The lower chamber contained serum-containing media. The plates were incubated for four days in triplicate, fixed, and stained with crystal violet, the underside was imaged, and the cells were counted with ImageJ.

### 2.7. Immunohistochemistry

p16-positive oropharyngeal cancers from never-smokers (n = 7) and smokers (n = 9) were identified following a review of pathology records and medical records. Sections of 7 mM were cut, dewaxed, and subject to 10 mM citrate pH 6 antigen retrieval in a 95 °C water bath for 20 min, and then cooled for 30 min in PBS, permeabilised with 0.5% Tween 20 PBS for 15 min, blocked sequentially with 10% goat serum 1% BSA and avidin/biotin (BioLegend 927301, San Diego, CA, USA), and incubated with CD44 (1/400; Cell Signaling 3570, Danvers, MA, USA) or ALDH1 (1/400; Abcam ab52492, Cambridge, UK) overnight. Next, sections were incubated with a secondary biotinylated antibody (1/800 anti-mouse, Sigma Aldrich B7401; 1/800 anti-rabbit Sigma Aldrich B8895) for 30 min and HRP Avidin D (Vector Labs, A-2004, Newark, CA, USA), and visualised with DAB (SignalStain^®^ DAB Substrate kit; Cell Signaling 8059). For a negative control, sections were treated as above but without primary antibody. Slides were counterstained with haematoxylin and DPX-mounted (Sigma Aldrich 06522). Images were taken on a Zeiss AXIO Scope, processed in ZEISS Zen lite software, and independently evaluated by LK.

### 2.8. RNA Extraction and Next Generation Sequencing

The Isolate II RNA Mini Kit (Bioline, London, UK) was used to purify RNA, purity was checked with an Agilent TapeStation (Santa Clara, CA, USA), and high-throughput Illumina sequencing was performed (The Australian Genome Research Facility). Sequence data were generated by NovaSeq Control Software (NCS) v1.7.0, Real Time Analysis (RTA) v3.4.4 and Illumina bcl2fastq 2.20.0.422. A total of 32 to 57 million paired-end reads were acquired for each sample, and raw files were checked by FastQC v0.11.7. and MultiQC v1.9. Read alignment was performed with Bowtie2 within RSEM v1.3.1 for the Human Transcriptome GRCh38p.13 (NCBI) and all samples passed QC and were mapped successfully (94–96%).

### 2.9. Bioinformatics

DESeq2 version 1.30.0 was used for normalisation and differential expression analysis was undertaken in pair-wise comparisons between the groups. Statistical significance (*p* < 0.05) was determined by the Wald test with a log2-fold change threshold of log2(1.25), with at least a 25% change in gene expression. DESeq2 was also used to normalise read counts based on sequencing depth and RNA composition using a median ratio method. Pathway enrichment was performed on the list of genes ranked by the Wald “stat” value; the Gene Ontology Enrichment Analysis and Visualization (GORILLA) tool identified pathway enrichment of GO biological processes, and the results were submitted to Reduce and Visualise Gene Ontology (REVIGO) to remove redundant pathway results. Heatmaps were generated through choosing genes based on the curated genetic pathways, with expression values generated from DESeq2′s variance-stabilising transformation and subtracting the mean expression from each value.

### 2.10. Flow Cytometry

Treated cells were detached with Accutase^®^ (Sigma Aldrich) and stained with anti-CD44 (1/100; clone IM7 APC Cy7 Rat IgG2b,κ BioLegend, 103028), Aldeflour™ (1/100; STEMCELL Technologies, 01700, Vancouver, Canada) and 7-aminoactinomycin D (1/100; BioLegend, 420404) for 30 min. Flow cytometry was performed on a BD LSRFortessa and analysed with FlowJo. A total of 10,000 events were recorded for each treatment group. Diethylaminobenzaldehyde (DEAB, ALDEFLUOR^TM^ Kit, Stem Cell Technologies) and an IgG isotype antibody (1/100; clone RTK4530 APC Cy7 Rat IgG2b,κ BioLegend, 400624) were used as a negative control for gating. All treatment groups were stained with 7-AAD (7-aminoactinomycin D; 420404, BioLegend) to identify non-viable cells. Images and analyses were derived with FlowJo^TM^ (version 10.10).

### 2.11. Ethics

This study was performed in concordance with the NHMRC National Statement on Ethical Conduct in Human Research (NHMRC 2007, updated 2018). Approval for this study was granted under HREC/18/QTHS/141 and LNR/2018/QTHS/45080. Identified patients were retrospectively recruited. Smoking history, alcohol use history, and non-indigenous and indigenous status were verified at the time of recruitment when the patient was alive. The recruitment spanned 17/8/18 to 9/11/20. In the case of deceased patients, a public health waiver was obtained to access their tissue specimen. For publication, all patient information was de-identified.

### 2.12. Statistical Analyses

Data are presented as mean ± standard error, and were compared using one-way ANOVA and Tukey’s multiple comparison tests. Statistical analysis was performed with GraphPad Prism (Version 8.3) and significance was defined as *p* < 0.05.

## 3. Results

### 3.1. Cigarette Smoke and Radiation Alter FaDu Cell Phenotype and CSC Number

**Figure 1:**
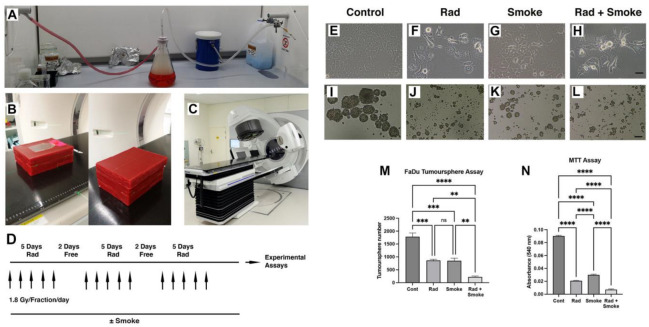
Radiation and cigarette smoke altered cell phenotype and reduced CSC number and cell proliferation. Vacuum apparatus used for generating smoke-containing media (**A**). Setup used for irradiating cells; open and complete wax block with culture flask (**B**). Elekta linear accelerator (**C**). The fractionated radiation protocol: FaDu cells with and without smoke exposure were radiated with 1.8 Gy 5 times per week for 3 weeks (**D**). Images of the control cells and those treated with radiation (Rad), smoke (Smoke), and RS (Rad + Smoke) (**E**–**H**; scale bar 50 µm) and tumour spheres (**I**–**L**, scale bar 100 µm). Tumour sphere assay: numbers represent average sphere number per treatment group (**M**) (n = 3). After 48 h, proliferation assays showed that radiation-, smoke-, and RS-treated cells had reduced proliferative capacity versus control (**N**); ** *p* < 0.01; *** *p* < 0.001; **** *p* < 0.0001; ns = not-significant.

To understand how radiation, cigarette smoke (DMEM media exposed to cigarette smoke), and the combination of radiation–cigarette smoke (RS) affects FaDu cells, cells were exposed to radiation at a clinically relevant radiation dose fractionation, cigarette smoke, or the combination of RS (Figure 1A–D). Notably, after treatment in standard cell culture flasks, cells treated with radiation, smoke, and RS began to lose their epithelial morphology and were less adhered to the culture dish (Figure 1E–H). To assess cancer stem cell (CSC) number, we performed tumour sphere assays. Radiation and cigarette smoke either alone or in combination resulted in the formation of smaller and fewer tumour spheres. Exposure to radiation or cigarette smoke alone halved the number of spheres formed, while the combined RS treatment reduced sphere formation 8-fold (Figure 1I–M). Proliferation assays demonstrated a dose–response effect of smoke on FaDu cells (Figure S1E), and that radiation-, smoke-, and RS-treated cells had greatly reduced growth of 4.3-, 3-, and 12.7-fold, respectively, compared to untreated cells (Figure 1N).

In comparative assays with FaDu and SCC15 cells, we observed that SCC15 cell proliferation was not affected by radiation but tumour sphere number was significantly reduced (Figure S1A–C). Furthermore, compared to Fadu cells, SCC15 cells did not exhibit a dose–response to media containing cigarette smoke and the cell morphology was unchanged (Figure S1D–F). Thus, for the remainder of the study we focussed on Fadu cells.

### 3.2. Cigarette Smoke and Radiation Promote an Aggressive Cell Phenotype

**Figure 2:**
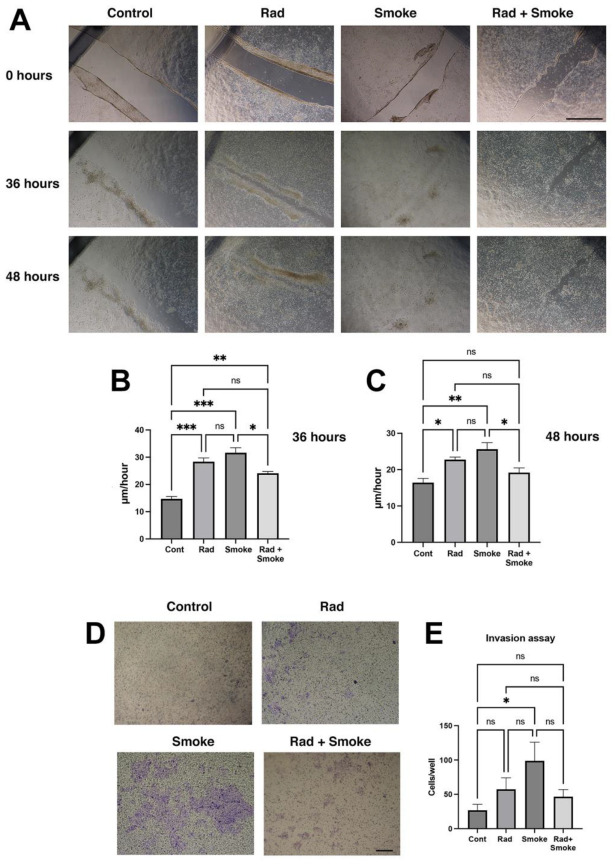
Radiation and cigarette smoke promote migration, wound healing and invasion. Migration assay: control and radiation-, smoke-, and RS-treated cells were allowed to grow to confluency and wounded, or subject to an invasion assay as described in the methods. Images were taken at 0, 36, and 48 h (**A**), and the wound gap was measured. The rate of healing calculated at µm/hour (**B**,**C**); scale bar 1000 µm. Invasion assay: after 4 days, more cells (blue) were on the underside of the membrane in treated cells (**D**). Quantification demonstrated a non-significant increase in radiation and RS, and a significant 4-fold increase in invasive cells in the smoke-exposed group (**E**); * *p* < 0.05; ** *p* < 0.01; *** *p* < 0.001; ns = not-significant; scale bar: 250 µm.

The change in cell morphology and tumour sphere characteristics suggested that the radiation-, smoke-, and RS-treated cells may have acquired new cellular properties. To confirm this, the control and treated cells were subjected to a wound-healing assay. Radiation and smoke treatments accelerated wound healing after 36 h by 2- and 2.2-fold, respectively, and RS by 1.7-fold. At 48 h, the radiation-, smoke-, and RS-treated cells had near-completed wound healing, whereas the control cells did not (Figure 2A–C). To further confirm that the cells had greater migratory ability, the cells were subjected to an invasion assay through Matrigel, a basement membrane matrix. Radiation and RS caused non-significant increases in FaDu cell invasion, whilst cigarette smoke alone significantly promoted cell invasion (Figure 2D,E). Together, the data suggest that FaDu cells subjected to radiation, smoke, and RS acquire migratory and invasive features.

### 3.3. Radiation and Cigarette Smoke Reduce CD44 and ALDH Co-Expression

**Figure 3:**
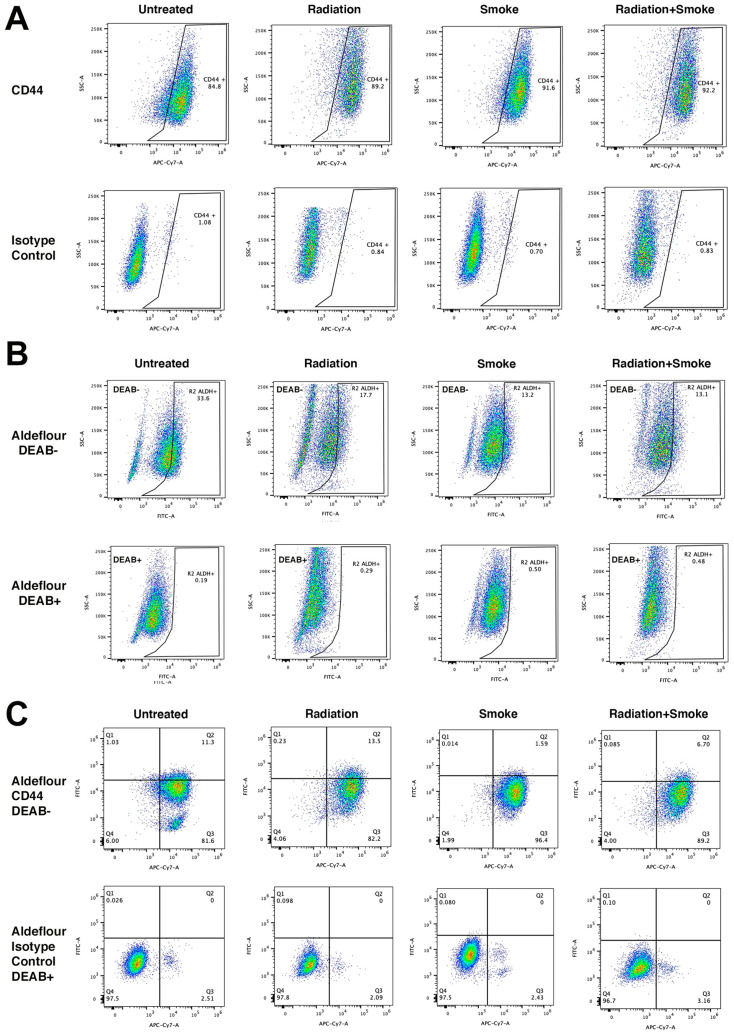
Radiation and cigarette smoke reduce CD44 and ALDH co-expression. Control radiation-, smoke-, and RS-treated cells were stained for the CSC markers ALDH and CD44. Data represent the cell population percentages of 10,000 events for control, radiation, smoke, or RS treatments for (**A**) single-stained with CD44 (APC-Cy7), (**B**) single-stained with ALDEFLOUR™ (FITC), and (**C**) dual-stained with ALDEFLOUR™ (FITC) and CD44 (APC-Cy7). For the ALDEFLOUR™- and CD44-negative control, FADU cells were stained with the ALDEFLOUR™ inhibitor DEAB and an IgG isotype control antibody, respectively. Table 1 lists the percentages of positive cells.

We determined the effect of radiation, smoke, and RS on the expression of the CSC markers CD44 and ALDH. Treated cells were stained with an antibody against CD44 and with ALDEFLUOR^TM^ reagent to determine ALDH activity. Flow cytometry was subsequently performed to quantify the populations of cells expressing these markers. Compared to controls, radiation, smoke, and RS increased the frequency of CD44-expressing cancer cells 1.05-, 1.08-, and 1.09-fold, respectively (Figure 3A). In contrast, ALDH activity was reduced in cells exposed to radiation, smoke, and RS 1.90-, 2.55-, and 2.56-fold, respectively (Figure 3B). Cells that expressed both CD44^+^ and ALDH^+^ increased 1.19-fold after radiation but were reduced 7.11- and 1.70-fold after smoke and RS, respectively (Figure 3C, Table 1). These data show that smoke and RS do not increase the number of cells that have both CD44 expression and ALDH activity.

### 3.4. Patients Who Smoke Have Similar CD44 and ALDH1 Expression to Non-Smokers Prior to Treatment

**Figure 4:**
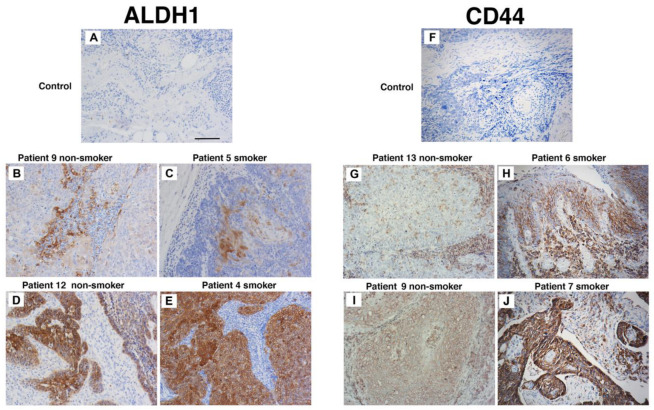
Smoking status does not affect CD44 and ALDH1 staining. Patient biopsy tissues specimens taken at diagnosis and prior to the commencement of treatment. Representative immunohistochemistry for ALDH1 (**A**–**E**) and CD44s (**F**–**J**) from non-smokers (patients 9, 10, 13, and 14) and smokers (4, 6, 7, and 11). Scale bar: 50 µm.

To evaluate the influence of cigarette smoke on the proportion of CSCs in untreated in vivo tumours, immunohistochemistry was used. We compared CD44 and ALDH1 expression in tumour biopsies from never-smokers (seven patients) and smokers (nine patients). Three indigenous patients were in the smoker group; the remaining thirteen were non-indigenous. These specimens were collected from patients with oropharyngeal SCC prior to the initiation of radiation therapy. As very few patients in our clinic are HPV-negative never-smokers, only HPV-positive tumours were used in this analysis. The patient details and immunohistochemistry outcomes are listed in Table 2. Most patients were male and aged between 47 to 74 years. Alcohol intake was significant for seven patients. Considering the groups, ALDH1 tumour cell staining was found in four smokers and five never-smokers (Figure 4A–E). In contrast, CD44 staining was present in three smokers and in five never-smokers and localised to tumour cells and in some instances to the tumour stroma (Figure 4F–J). Importantly, strong staining of both ALDH1 and CD44 did not occur in the same patient. These data suggest that cigarette smoke alone does not increase the expression of CD44 and ALDH1 and supports our in vitro findings that cigarette smoke does not increase CSC number.

### 3.5. Cigarette Smoke and Radiation Promote a Specific Gene Signature

Considering that: (i) all treatments induced a more aggressive phenotype and decreased CSC number, (ii) smoke and RS reduced CD44 expression and ALDH activity, and (iii) in patients, cigarette smoke does not increase CD44 and ALDH1 expression, we undertook next generation sequencing to identify genetic differences and pathways induced by radiation, smoking and RS.

From the 12 samples, gene expression was quantified for 469,668 transcripts and principal component analysis showed that distinct groups represented control, radiation, smoke, and RS (Figure S2A). Versus control cells, 4051 differential expressed genes (DEGs) were expressed in radiation, 652 in smoke, and 4668 in RS. Of the DEGs in RS cells, 65.4% were found in the radiation group, 10.2% in smoke treatment, and 21.3%, were unique to RS (Figure S2B,C). In pathway enrichment analyses, the treatments when compared to controls, promoted genes representing functions in cell motility, migration, locomotion, cell-cell adhesion, immune response, cell signalling and angiogenesis (Figure S3A,C,E). In contrast, the down-regulated pathways in all treatments represented cell division and cell death (Figure S3D,F; and Table S1: normalised gene expression data; and sequencing data have been assigned the GEO number GSE224803).

**Figure 5:**
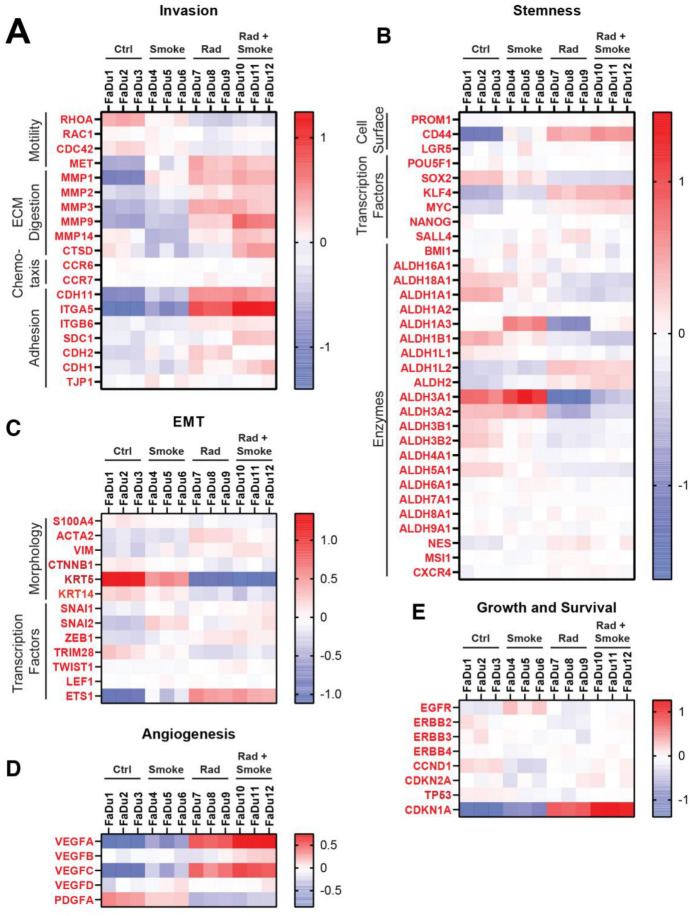
Radiation and cigarette smoke increase the expression of several genes with roles in EMT and stem cells. Using next generation sequencing, the gene expression profiles of FaDu cells exposed to radiation, smoke, or RS were compared to nontreated control FaDu. The heatmaps show the changes in expression for selected genes involved in metastasis under the groups of invasion (**A**), stemness (**B**), EMT (**C**), angiogenesis (**D**), and growth and survival (**E**).

To determine if treatment affected aggressive cellular pathways, genes were chosen from publications that identified markers of invasion, stemness, endothelial-mesenchymal transition (EMT), angiogenesis, growth and survival, and heatmaps were generated [24,25,26,27,28]. Alterations in invasion markers were variable (Figure 5A). On note, the cellular adhesion molecules CDH11 and ITGA5 were increased after radiation, and ITGA5 was further upregulated after RS. Interestingly, matrix metalloproteinases (MMP2, MMP9, and *MMP14*) and cathepsin D (CTSD) were upregulated after RS. RHOA, a motility-related gene, was decreased after radiation and RS, which contrasts with the observed increased migration of these cells in vitro.

In agreement with the reduction in tumour sphere number after the treatments, stem cell markers showed mostly small and variable changes. CD44 was increased after radiation and RS. Except for ALDH1L2 and ALDH2, the remaining ALDH isoforms were decreased after radiation and RS. KLF4 and MYC were upregulated in the RS group (Figure 5B). Limited change was observed in genes representing EMT (Figure 5C), with the exception that KRT5 was downregulated after radiation and RS. Smoke increased ETS1, a transcription factor implicated in EMT, and it was further elevated after radiation and RS. The angiogenesis-related genes VEGFA/B/C were increased after radiation and RS and PDGFA was downregulated in all groups (Figure 5D). There were limited changes in genes representing growth and survival (Figure 5E), with only radiation and RS causing increases in CDKN1A.

## 4. Discussion

Curative treatment for mucosal HNSCC involves surgery and/or radiation therapy, with or without concurrent chemotherapy [29]. The purpose of radiation therapy is to generate DNA damage in tumour cells, via free radical production, leading to cell death. Unfortunately, many head and neck cancer patients smoke, which is a key risk factor for the development of these cancers. Moreover, continuing to smoke during and after radiation treatment results in worse oncological and normal tissue toxicity outcomes. These include increased recurrence rates, worse disease-free and overall survival, dysphagia, increased likelihood of gastrostomy tube dependence, severe induration and fibrosis of subcutaneous tissues, laryngeal oedema, and oesophageal strictures [8,30,31]. Many patients often find smoking cessation particularly difficult during the stressful events of diagnosis and treatment, and hence smoking cessation is not easily achieved during treatment. Despite these facts, there is little known about the cellular events and molecular pathways that underpin these poor outcomes due to a lack of in vitro models to investigate them.

To the best of our knowledge, this is the first report that has examined the combined action of radiation and cigarette smoke (RS) on mucosal HNSCC cells. We find that smoke-exposed medium by itself or in combination with radiation did not increase tumour sphere number or cell proliferation in vitro. This is consistent with the decreased co-expression of CD44 and ALDH in flow cytometry, and variable effects on other CSC markers/reprogramming factors as observed through our bioinformatic analyses. Given that smokers have worse prognosis, the findings of reduced CSCs were unexpected. It is possible that there are other subpopulations of CSCs responsible for the poor prognosis in smokers and there is no pan marker to identify these, and that longer culture cycles are required to support the survival of these CSCs.

Our results illustrating that radiation alone increases co-expression of CD44^+^ and ALDH are consistent with the work of Reid and colleagues [11]. They used UM-SCC-1 and UM-SCC-47 cells and delivered a limited number of large doses of radiation per treatment and saw increased CD44^+^ ALDH activity versus untreated cells. Separately, Vlashi et al. showed that a single radiation dose could promote the de-differentiation of HNSCC HPV negative cells into CSCs [10]. In our hands, radiation reduced the FaDu tumour sphere number versus the control, suggesting that these observations are cell-line-specific, or influenced by dose delivered per fraction.

All three treatments in our study increased migratory and invasive capacity. Next generation sequencing supported this as radiation and RS treatments increased the expression of cell adhesion (ITGA5), MMPs (MMP2, MMP9, and MMP14), and angiogenic proteins (VEGFA and VEGFC). Moreover, ITGA5 is a potential biomarker [32] and correlates with worse survival [33] and VEGF is upregulated in oral SCC and parallels with invasion and poor survival [34,35]. Foki et al. has similarly shown integrins, MMPs and VEGFA to be upregulated by human oral keratinocytes exposed to cigarette smoke [36]. For EMT related genes, the changes were less dramatic. KRT5 and KRT14 were decreased in cells exposed to smoke, radiation and RS. These proteins appear to influence EMT gene expression in a complex pattern that is not fully understood [37,38]. Similarly, ETS1 was upregulated after radiation and RS and is a regulator of EMT and cell invasion [39]. Further work is required to validate the association of RS with EMT induction.

HPV and smoking are risk factors for mucosal HNSCC development. ALDH1 is prognostic with respect to disease control and distant metastases in patients independent of HPV status [18]. For CD44 and regardless of HPV status, studies show conflicting results concerning CD44’s association in HNSCC. By example, studies using immunohistochemistry, show that reduced CD44 expression, predicts reduced survival [40,41]. Whereas, others show that CD44 is consistently increased on all head and neck tissues, from normal to HNSCC [42]. When examined in vivo, we found that CD44 and ALDH1 did not co-express in the same patient or associate with smoking status or alcohol intake. This differs from the findings of Kokko et al., who found that heavy smoking is associated with tumour CD44 overexpression in HNSCC. Whilst this may be due to the small population size of our data, it may also be due to the population studied. Kokko et al. focussed on a Finish population with unknown HPV status, unlike our data which arose from Caucasians of Anglo-Saxon origin and indigenous Australians that were HPV-positive [43]. Furthermore, as our investigation consisted of just 16 patients, any future work should involve a larger patient cohort and patients with HPV-negative status.

Although compelling, there are limitations to our study. While the sphere-forming assay allows growth and propagation of CSCs, it has limitations pertaining to the assessment of the number and size of spheres. Additionally, as we saw indifferent data with SCC15 cells, we focussed only on FaDu cells; thus, it is important that future studies consider other HNSCC cell lines, the HPV status, and eventually the use of patient-derived tumour xenografts to validate the roles of HPV, smoking, and radiation in tumour progression. The markers used in our study may also not be sufficient to precisely isolate CSC subpopulation and other markers should be considered to define and isolate these [44]. Additionally, different CSC subpopulations may exist in the same tumour [45].

## 5. Conclusions

In summary, we have established a new experimental approach to evaluate the combined effects of fractionated radiation and smoking on HNSCC cells. We found that radiation and cigarette smoke increase the migratory and invasive capacity of cancer cells. Future studies will involve broadening the scope of the cellular systems and characterising the function of specific genes in HNSCC tumourigenesis.

## Figures and Tables

**Table 1:** The percentages of CD44- and Aldeflour-stained cells after each experimental condition.

	Control	Rad	Smoke	Rad+ Smoke
CD44	84.8	89.2	91.6	92.2
Aldeflour	33.6	17.7	13.2	13.1
Aldeflour + CD44	11.3	13.5	1.59	6.70

**Table 2:** Patient details, tissue, pathology, and IHC results.

Patient Number	Smoking Status	Packs/Year	Alcohol Intake	Sex/Age	HPV Status	Tissue Type	Pathology	**ALDH IHC**	**CD44 IHC**
1	Yes	<10	Heavy	M 69	+	Right tonsil	SCC; not keratinised	0	0
2	Yes	27	Medium	M 64	+	Tumour left tongue base	SCC; dysplastic; infiltrating; not keratinised	0.5	0
3	Yes	50	Nil	M 73	+	Right tonsil	SCC, non-keratinising; basaloid	0	0
4	Yes	50	Heavy	M 59	+	Left tonsil	SCC; non-keratinised	4	0
5	Yes	47	Heavy	M 68	+	Right tonsil base extending to posterior border of tongue	SCC; keratinised	1	4
6	Yes	50	Medium	M 71	+	Left extended tonsillectomy	SCC; non-keratinising and invasive	4	1
7	Yes	70	Heavy	M 59	+	Left tonsil	SCC; keratinised with keratin pearls	0	4
8	No	0	Rare	M 63	+	Left base of tongue	SCC, keratinised	0	0
9	No	0	Low	M 55	+	Right tonsil	SCC, keratinised	1	1.5
10	No	0	Minimal	F 68	+	Right tonsil	SCC: non-keratinised; spindel cells	0	1
11	No	0	Low	M 63	+	Right tonsil	SCC, focal keratinisation in regions	1	1
12	No	0	Heavy	M 74	+	Right tonsil	SCC, keratinised	4	1
13	No	0	None for 10 years	M 61	+	Right tonsil	SCC; non-keratinised	2.5	0
14	No	0	Rare	F 47	+	Left tonsil	SCC; non-keratinised	1	0
15	No	0	Rare	M 69	+	Left tonsil	SCC; non-keratinised	0	0
16	No	0	Low	M 49	+	Right glossotonsilar	SCC; non-keratinised	0	1

## Data Availability

All data generated or analysed during this study are included in this published article (and its Supplementary Information Files). Bioinformatic data have been assigned the GEO number GSE224803 (submission date 8 February 2023) and can access the data through the link https://www.ncbi.nlm.nih.gov/geo/query/acc.cgi?acc=GSE224803.

## Supplementary Materials

The following supporting information can be downloaded at https://www.mdpi.com/article/10.3390/cancers17081346/s1: Figures S1–S3: Bioinformatic information. Table S1: Sequencing data Excel file.

## Abbreviations

The following abbreviations are used in this manuscript:

HNSCCs	Head and neck squamous cell cancers
ALDH	Aldehyde dehydrogenase
HPV	Human Papilloma Virus
