# Supplementary material for: A New Model to Investigate the Action of Radiation and Cigarette Smoke on Head and Neck Cancer Cells
Source: Cancers (Basel). 2025 Apr 17;17(8):1346. doi: 10.3390/cancers17081346 (PMC12026225; doi:10.3390/cancers17081346)
Supplement: Supplementary file 1 [file cancers-17-01346-s001.zip › supplementary figures S1-S3.pdf]

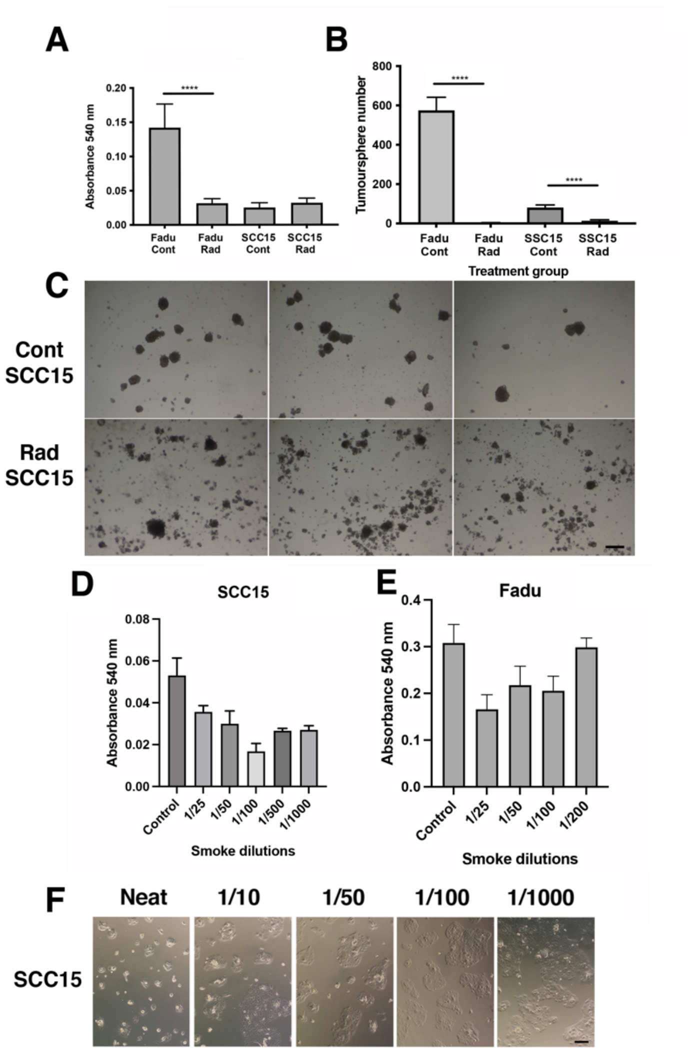

Figure S1. Comparative effects of radiation and cigarette smoke on Fadu and SCC15 cells.

FaDu and SCC15 cells were treated with a cumulative dose of 27Gy and 23.4Gy of radiation, and subjected to proliferation (A), and tumoursphere (B) assays. After radiation, Fadu cell proliferation and tumoursphere number was reduced, and SCC15 cells showed only reduced tumoursphere number. Images of SCC15 control (Cont) and radiated (Rad) treated cells; scale bar 50 μm (C). FaDu and SCC15 cells were grown in dilutions of smoke containing media in growth media and subjected to proliferation assays. After 48 hours, SCC15 cells did not elicit a dose-response effect, whereas Fadu cells did (D-E). Images of SCC15 cells after smoke treatment in dilutions of smoke containing growth media; scale bar 25 μm. **** $p<0.0001$.

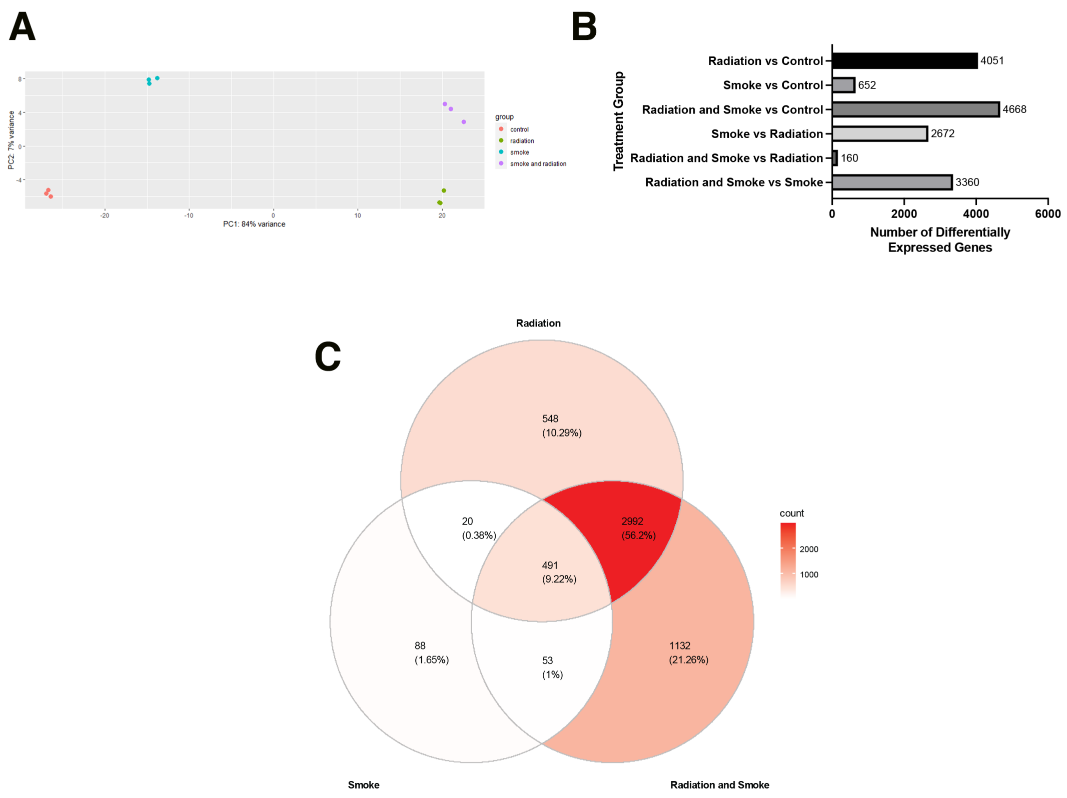

Figure S2. Bioinformatic analyses 1.

Principal component analyses show that the gene expression data of each treatment group forms tight groups (A). Differential gene expression between treatment and control for changes of greater than 25% found distinct numbers of genes for each comparison (B). Venn diagram of the three treatment groups showing association with differential gene expression for each or combined (C).

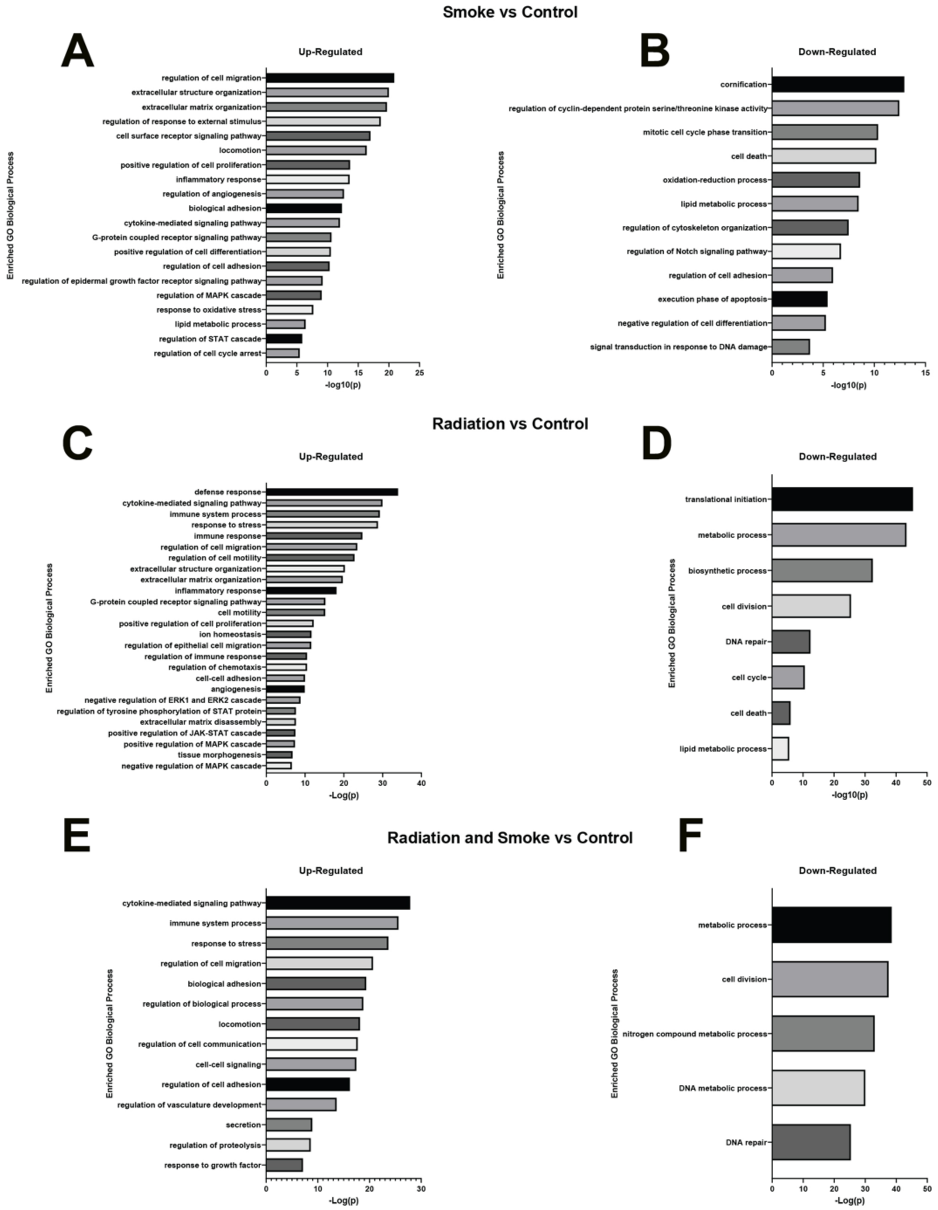

Figure S3. Bioinformatic analyses 2.

Pathway enrichment: Radiation and smoke treatment compared with control cells. Differential expression was performed on cells treated with smoke (A) versus control (B); radiation (C) versus control (D); and RS (E) versus controls (F) using DESeq2. Pathway enrichment was performed using GORILLA.
